# Salivary peptidome profiling for diagnosis of severe early childhood caries

**DOI:** 10.1186/s12967-016-0996-4

**Published:** 2016-08-15

**Authors:** Xiangyu Sun, Xin Huang, Xu Tan, Yan Si, Xiaozhe Wang, Feng Chen, Shuguo Zheng

**Affiliations:** 1 Department of Preventive Dentistry, Peking University School and Hospital of Stomatology, National Engineering Laboratory for Digital and Material Technology of Stomatology, Beijing Key Laboratory of Digital Stomatology, 22 Zhongguancun Avenue South, Haidian District, Beijing, 100081 People’s Republic of China; 2 Stomatological Hospital of Guizhou Medical University, Guiyang, 550004 People’s Republic of China; 3 Central Laboratory, Peking University School and Hospital of Stomatology, National Engineering Laboratory for Digital and Material Technology of Stomatology, Beijing Key Laboratory of Digital Stomatology, 22 Zhongguancun Avenue South, Haidian District, Beijing, 100081 People’s Republic of China

**Keywords:** MALDI-TOF MS, Proteomics, Saliva, s-ECC, Biomarker, Histatin-1, Early diagnosis

## Abstract

**Background:**

Severe early childhood caries (s-ECC), which has quite high prevalence among children, is a widespread problem with significant impacts among both developing and developed countries. At present, it is widely known that no early detective techniques and diagnostic tests could have high sensitivity and specificity when using for clinical screening of s-ECC. In this study, we had applied magnetic bead (MB)-based matrix-assisted laser desorption/ionization time-of-flight mass spectrometry (MALDI-TOF MS) to screen distinctive candidate biomarkers of this disease, so as to establish protein profiles and diagnostic models of s-ECC.

**Methods:**

Firstly, we used the technique mentioned above to detect specifically expressed peptides in saliva samples from ten children with s-ECC, separately at the time point of before, 1 and 4 weeks after dental treatment. Then a diagnostic model for s-ECC was established with the K nearest-neighbour method, which was validated in another six children in the next stage of study. After that, linear ion trap-orbitrap-mass spectrometry (LTQ-Orbitrap-MS) was performed to identify which of the proteins in saliva might be the origination of these peptides.

**Results:**

We found that seven peptide peaks were significantly different when comparing the three time points, among them two were higher, while other five were lower in the pre-treatment s-ECC group compared with post-treatment. The sensitivity and specificity of the diagnostic model we built were both 83.3 %. Two of these peptides were identified to be segments of histatin-1, which was one important secretory protein in saliva.

**Conclusions:**

Hereby we confirmed that MB-based MALDI-TOF MS is an effective method for screening distinctive peptides from the saliva of junior patients with s-ECC, and histatin-1 may probably be one important candidate biomarker of this common dental disease. These findings might have bright prospect in future in establishing new diagnostic methods for s-ECC.

**Electronic supplementary material:**

The online version of this article (doi:10.1186/s12967-016-0996-4) contains supplementary material, which is available to authorized users.

## Background

Dental caries is one common chronic disease among children [1], remaining a major problem in many countries [2–5]. Severe early childhood caries (s-ECC) is its aggressive form, defined by one or more of the following criteria: (1) any sign of smooth surface caries in children aged <3; (2) one or more decayed, missing (due to caries), or filled tooth surfaces in primary maxillary anterior teeth in those aged 3–5; (3) having decayed, missing, or filled surface score (dmfs) more than four at age 3, or more than five at 4, or more than six at 5 [6]. s-ECC can reduce the children’s ability of eating and drinking, resulting in tooth decay or malocclusion of permanent dentition [7, 8]. These further cause malnutrition, speaking and sleeping problems, as well as impair the school performance, social behaviour, and self-esteem of the patients [4]. Therefore, s-ECC has made many negative impacts on the quality of life of preschool children and their parents [8, 9].

Paediatric dental caries is also a significant problem in China. According to the national epidemiological report, the prevalence of dental caries among 5-year-old children reached 66 %, with mean dmfs 3.5 [10]. Recent studies demonstrated that polarization of caries has been more pronounced [11–13].

The known factors influencing dental caries in children includes: (1) immature immune systems; (2) characteristics of saliva; (3) cariogenic microorganisms; (4) feeding type; (5) oral hygiene care in early childhood [14]. Recently, the characteristics and components of saliva have also been suggested to forewarn the development of s-ECC [15].

Saliva is one important body fluid containing complex components which has great diagnostic value [16, 17]. As the host-associated factor, saliva plays an essential role in dynamic equilibrium between demineralization and remineralization, changing the sensitivity and resistance to dental caries [18]. Many proteins in saliva function during adhesing and aggregating of cariogenic bacteria [18–20]. However, some other proteins in saliva may contribute to the defence against microorganisms [21–23].

Recently, new proteomics techniques, including high-throughput analysis of the salivary proteome, make it possible to characterize its protein composition and investigate its impact on development of dental caries. Matrix-assisted laser desorption/ionization time-of-flight mass spectrometry (MALDI-TOF MS) is a useful method for identification of microbes and proteins, which has high efficiency and automation but lower costs [24–26], making great benefits to clinical and laboratorial experiments. It is one of the most promising techniques to reduce the huge gap between high throughput proteomics and clinical studies with large samples [27, 28]. MALDI-TOF MS can be used in combination with weak cation-exchanger (WCX) magnetic bead to select peptides from saliva in the range of 1000–10,000 Da prior to further identification. The WCX magnetic beads can separate the proteins and/or peptides of different isoelectric points from complex biological fluids with specific anionic ligands, which is confirmed to be suitable for the fractionation of low-molecular-mass range peptides (1–10,000 Da) and can exhibit optimal performance which can be confirmed in many studies, such as some cancer research [29, 30]. The techniques of MALDI-TOF MS combined with WCX magnetic beads incorporate both of their advantages such as low cost and simple purification, which could capture more peptides than some other methods especially in the low-molecular-mass range [31, 32].

In the previous study from our research group, we have designed a cross-sectional study to introduce weak cation-exchanger magnetic bead-based matrix-assisted laser desorption/ionization time-of-flight mass spectrometry (WCX MB-based MALDI-TOF MS) to compare the components of salivary peptides in children with and without s-ECC, and successively found certain differentially expressed peptides between caries and non-caries children [33]. Based on the former research, in the present study we also use this method, WCX MB-based MALDI-TOF MS, to evaluate the profiles of salivary peptides in those children with s-ECC at different time points of before, 1 and 4 weeks after treatment, so as to verify the hypothesis that certain peptides in saliva are associated with different periods of before and after treatment of dental caries in children, and could be used as candidate biomarkers for diagnosis and identification of status of s-ECC.

## Methods

### Ethics statement

This study was ethically approved by Institutional Review Board of Peking University School of Stomatology (PKUSSIRB-2013060). The parents of children who attended this study have all signed informed consent.

### Sampling

The sampling for this study was performed at the kindergarten affiliated to Renmin University of China (Beijing, China) in late 2013. Only children with the following conditions were included: (1) 3–5 years old, clinically diagnosed with s-ECC; (2) no influenza or infection of respiratory system, and received neither antibiotic therapy within 1 month nor fluoride prophylaxis within 1 year; (3) no medical history of congenital diseases; (4) able to spit the saliva and comply with dental treatment; (5) informed consent signed by the parents. 16 children reaching these criteria were included in the study.

### Collection and processing of saliva samples

After breakfast, all these children rinsed their mouth with clean water, then their stimulated whole-saliva samples were collected. After centrifugation at 10,000×*g* for 10 min at 4 °C, the supernatant was obtained, and 1 mM ethylene diamine tetraacetic acid (Sigma, St. Louis, MO) together with 1 mM phenylmethylsulfonylfluoride (Sigma) were added to inhibit protease activity. Protein concentration was measured by Lowry method and ELx808 Protein Assay (BioTek, Hercules, CA). Then these supernatants were stored at −80 °C.

### Pretreatment of MBs

A weak cation exchange magnetic bead (WCX MB) kit from Bioyong Tech (Beijing, China) was used. Alpha-cyano-4-hydroxycinnamic acid (CHCA) was dissolved freshly in 100 % ethanol (chromatographic grade) and 100 % acetone (chromatographic grade) to prepare the sample matrix for MALDI-TOF MS (Bruker Bio-sciences, Bremen, Germany).

All saliva samples were fractionated using WCX MBs (Bioyong Tech, Beijing, China). Samples were purified and isolated with the following steps: (1) 20 μL of beads, 150 μL of MB-WCX binding solution (CB), and 20 μL of salivary sample were mixed carefully and incubated for 5 min. (2) The tubes were placed on the MB separation device (Bioyong Tech) and the beads were allowed to collect on the tube wall for 1 min. (3) The supernatant was removed by washing and mixed thoroughly with 180 μL of MB washing solution (CW). (4) Another 10 μL of MB elution solution (CE) was added, and the beads were allowed to gather on the tube wall in the separation device for 2 min. (5) Clear supernatant was transferred into a fresh tube, and the peptides were analysed directly on a ClinTOF instrument (Bioyong Tech) or stored at −20 °C.

### Anchor chip spotting and MALDI-TOF MS profiling

The matrix solution, 5 mg/mL CHCA in 50 % acetone/0.1 % TFA solution (α-cyano-4-hydroxycinnamic acid) was prepared. First, 1 µL of purified peptide solution was spotted onto a MALDI-TOF MS target by ClinTOF (Bioyong Tech). After drying at room temperature, 1 µL of matrix solution was spotted onto the sample, and dried again before analysis. MALDI-TOF MS measurements were performed using a ClinTOF instrument (Bioyong Tech).

Before analysing, a three-peptide mixture (monoisotopic molecular weights of 1532.8582, 2464.1989, and 5729.6087 Da, Product Numbers P2613, A8346, and I6279, respectively; Sigma) was used for calibration of the MALDI-TOF MS. Profile spectra were acquired from an average of 400 laser shots per sample. The mass range of 1000–10,000 Da was collected. Each sample of saliva was analysed for 3 times, and the mean value of each sample was used for the analysis.

### Data processing

We chose ten children randomly from the full sample for analysis of salivary peptide profiles in each group (s-ECC before treatment, 1 and 4 weeks after treatment), and in total 30 salivary samples were analysed. The reproducibility of the mass spectra was determined from the mean relative peak intensities. All of the spectra obtained from the saliva samples in the training set were analysed using BioExplorer (Bioyong Tech) to subtract the baseline, normalize spectra (using total ion current), and determine peak *m*/*z* values and intensities in the mass range 1000–10,000 Da. A signal-to-noise ratio >5 was required. To align the spectra, a mass shift of no more than 0.1 % was determined. The peak area was used for quantitative standardization. The KNN in this software suite was used to establish the best pattern of diagnostic model for identifying s-ECC. Validation of the diagnostic model was carried out in a new series of the other six children with s-ECC, at the time points of before and after treatment for 1 and 4 weeks, and both sensitivity and specificity of this model were calculated. In this section, after each profile generated, 20 % leave-out cross-validation was performed in the software. Comparison of relative peak intensity levels between classes was also performed, and *P* <0.01 was considered as threshold of statistical significance.

### Identification of candidate peptide biomarkers by nano-LC/ESI–MS/MS

#### Peptide sequences

The sequences of diagnostic peptides in the model were identified using a nano-liquid chromatography-electrospray ionization-tandem mass spectrometry (nano-LC/ESI–MS/MS) system consisting of an ACQUITY UPLC system (Waters, Milford, MA) and an LTQ Obitrap XL mass spectrometer (Thermo Fisher Scientific, Rockford, IL) equipped with a nano-ESI source. The MS/MS experimental protocol was as follows: The peptide solutions were loaded onto a C18 trap column (Symmetry nanoACQUITY; Waters) (180 μm × 20 mm × 5 μm) with a flow rate of 15 μL/min. Then, the desalted peptides were enriched on a C18 analytical column (Symmetry nanoACQUITY) (75 μm × 150 mm × 3.5 μm) at a flow rate of 400 μL/min. Third, the mobile phases A (5 % acetonitrile and 0.1 % formic acid) and B (95 % acetonitrile and 0.1 % formic acid) were used on the analytical columns. Peptides were eluted using solvent B with the gradient elution profile 5 % B—50 % B—80 % B—80 % B—5 % B—5 % B in 100 min. The MS instrument was operated in data-dependent mode. The range of the full scan was 400–2000 *m*/*z* with a mass resolution of 100,000 (*m*/*z* 400). The eight monoisotope ions with the highest intensities were the precursors for collision-induced dissociation. MS/MS spectra were limited to two consecutive scans per precursor ion followed by 60 s of dynamic exclusion. Acetylization (N-Term) and oxidation (M) were used as post-translational modifications.

#### Bioinformatics and identification of candidate saliva biomarkers

The chromatograms were analysed with Bioworks Browser 3.3.1 SP1 and the resulting mass lists were used for database searches using Sequest™ (IPI Human 3.45). The parameters for generating the peak list were as follows: parent ion, 50 μg/g; fragment mass relative accuracy, 1 Da.

### Western blotting analysis

Salivary protein concentration was measured by bicinchoninic acid (BCA) kit (Solarbio, China). Salivary total protein (20 μg) underwent electrophoresis in 18 % gels for sodium dodecyl sulfate polyacrylamide gel electrophoresis (SDS–PAGE) using Mini-PROTEAN Tetra Cell System (Bio-Rad, USA). Proteins were then transferred to polyvinylidene fluoride (PVDF) membranes. The total protein load was checked visually by Ponceau staining before immuno-detection. The membranes were blocked overnight at 4 °C with 10 % skimmed milk in Tris-buffered saline (TBS, pH 7.4), followed by washing with TBS buffer containing 0.05 % tween20 (TBST). Membranes were incubated with the primary antibody specific for histatin-1 protein (ab70024, Abcam, UK) and anti-GAPDH (glyceraldehyde-3-phosphate dehydrogenase) monoclonal antibody (TA-08, ZSGB-Bio, China) at room temperature for 1 h. And the secondary antibodies used were rabbit polyclonal anti mouse immuno-globin G-horseradish peroxidase (IgG-HRP) (ab97046, Abcam) with the same condition, then the excess ones were removed. After that, the immuno-reactive bands were visualized by Odyssey scanner (Gene Company Limited, Hong Kong), and western blotting bands were quantified using Image J software (http://www.rsbweb.nih.gov/ij/).

### Statistical analysis

Data were analysed with the BioExplorer statistical package (Bioyong Tech). Depending on the results of normality tests, analysis of variance (ANOVA) or Kruskal–Wallis test was chosen to analyse statistical differences in peptide levels among saliva samples. The western blotting data was analysed with two-tailed paired t test. In all analyses, *P* < 0.01 was identified as statistical significance.

## Results

### Study population

In total 16 children with an average age of 4.7 ± 0.5 years (11 males and 5 females) received oral examination, and were sampled for saliva before and after 1 and 4 weeks of treatment. Information of these participants were shown in Additional Table 1 (Additional file 1).

### Peptide profiles

**Fig. 1:**
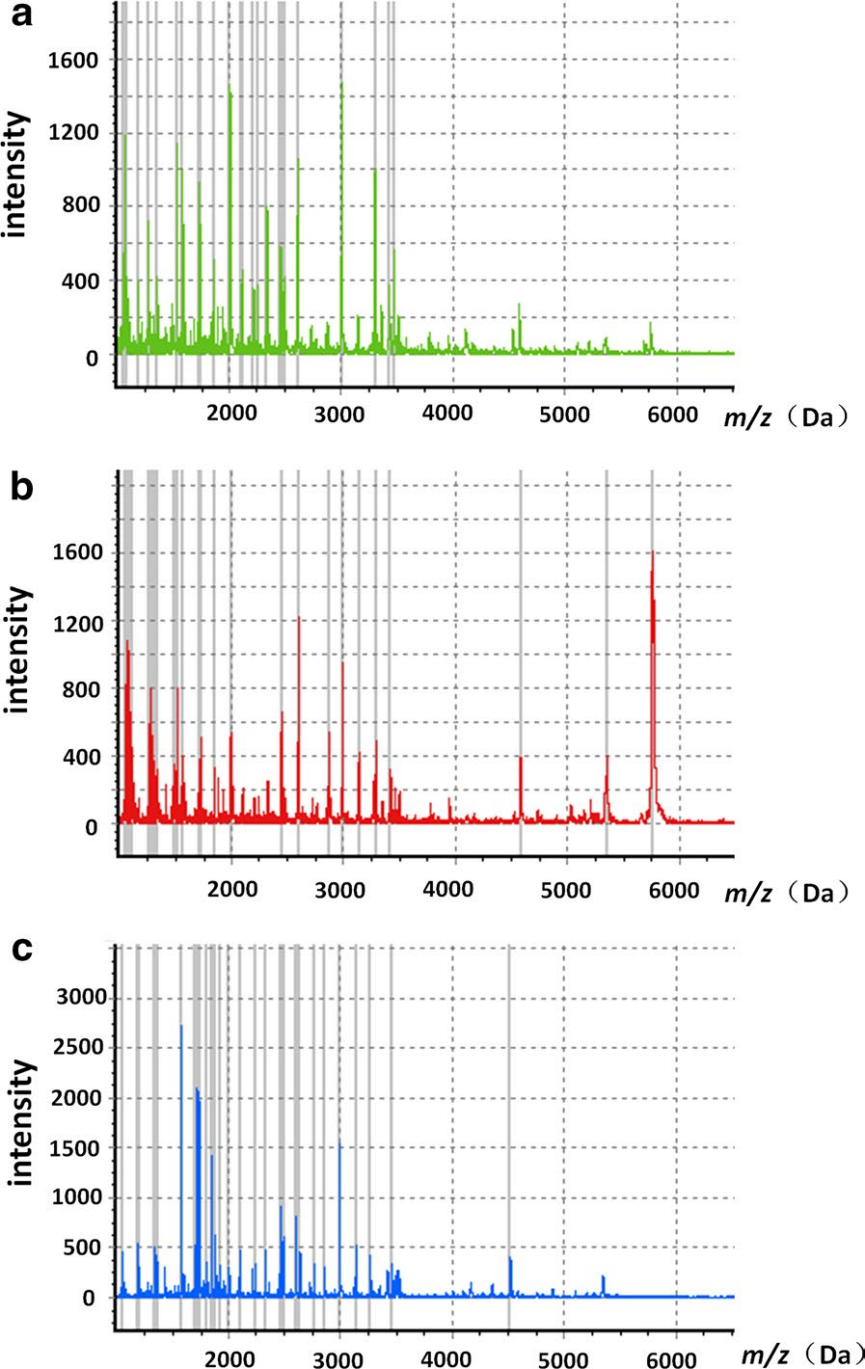
The entire mass spectra of the peptide samples in the range 1000–10,000 Da. These data were from ten patients with s-ECC before and after treatment. *Green*, untreated; *red*, treated for 1 week; *blue*, treated for 4 weeks. *m*/*z*, mass-to-charge ratio

30 stimulated whole saliva samples were collected from 10 children (randomly selected from the whole sample) with s-ECC before and after 1 and 4 weeks of treatment. The entire mass spectra of the peptides from these samples were analysed and compared by MALDI-TOF MS (Fig. 1). Saliva peptidome fingerprint peaks from each patient were quantified based on the maximum intensity within a particular *m*/*z* range. The majority of the peptides had molecular weights in the range 1000–7000 Da.

**Fig. 2:**
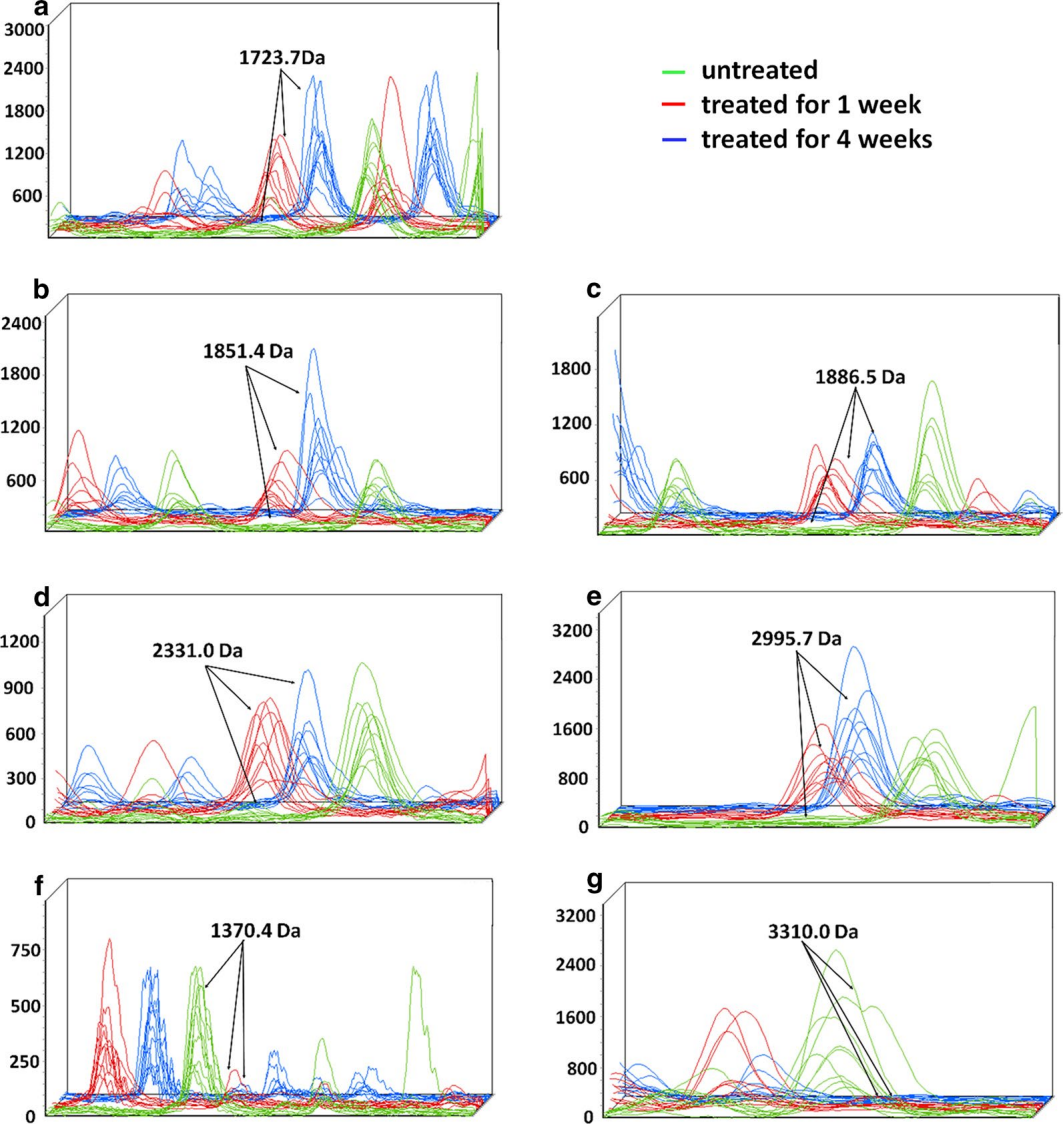
Three-dimensional *m*/*z* ratio-intensity maps. Those showed the seven significantly different peptides at 1723.7, 1851.4, 1886.5, 2331, 2995.7, 1370.4, and 3310.0 Da. *Green*, untreated; *red*, treated for 1 week; *blue*, treated for 4 weeks

**Fig. 3:**
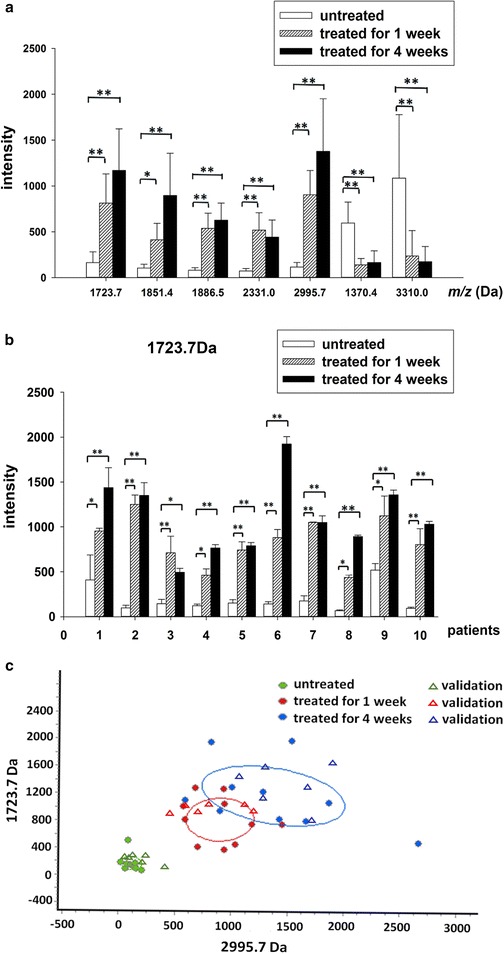
Histograms and *scatter plots* from the three groups. **a** Histogram of the mass spectra from the three groups, showing increased expression of peptides at 1723.7, 1851.4, 1886.5, 2331.0, and 2995.7 Da, and decreased expression of those at 1370.4 and 3310.0 Da, in s-ECC patients treated for 1 and 4 weeks. (*P < 0.05; **P < 0.01). **b** Histogram of the mass spectra at 1723.7 Da of all patients from the three groups, showing increased expression in s-ECC patients treated for 1 and 4 weeks. (*P < 0.05; **P < 0.01). **c** Scatter plots of the three groups established by combining the peptides at 1723.7 and 2995.7 Da, and showing well-distinguished curve fitting

Eighty one peptide mass peaks were simultaneously detected in all the 3 groups as shown in Additional Table 2 (Additional file 1). The peak intensities differed significantly for 7 peptides (experimental *m*/*z* values: 1370.4, 1723.7, 1851.4, 1886.5, 2331, 2995.7, and 3310.0 Da; Figs. 2, 3a), which abundance values were shown in Additional Table 3 (Additional file 1). Two peptides (experimental *m/z* values: 1370.4 and 3310.0 Da) were higher in the s-ECC children before treatment, whereas other 5 peptides (experimental *m/z* values: 1723.7, 1851.4, 1886.5, 2331.0, and 2995.7 Da) got higher peaks in the groups treated for 1 and 4 weeks.

Two peptides (experimental *m*/*z* values: 1723.7 and 2995.7 Da) exhibited the most significant difference (*P* < 0.01) when comparing these groups, while the fitted results of the other combinations were less good. In addition, these 2 peptides were downregulated in the untreated group (Figs. 2, 3a). The level of the peptide with experimental *m*/*z* values of 1723.7 Da was lower in each of the untreated children compared with after treatment for 1 and 4 weeks (Fig. 3b). Therefore, we chose these two peptides to establish a fitted curve for they could show the best distinction (Fig. 3c). The shape showed that the samples from the 3 groups were well-separated, indicating that the fitting results were satisfactory.

Four of the seven mass peaks (experimental *m*/*z* values: 1723.7, 1851.4, 2331.0, and 2995.7 Da) were used to establish a diagnostic model by the KNN method. In validation stage, five of the six s-ECC patients (sensitivity 83.3 %) were clinically diagnosed as s-ECC, and five of those treated for 1 and 4 weeks (specificity 83.3 %) were correctly grouped by this diagnostic model (Fig. 3c).

Moreover, with LTQ-Orbitrap-MS detection, four of the seven differentially expressed peptides (experimental *m*/*z* values: 1723.7, 1851.4, 1886.5, and 2331.0 Da) were successfully identified (Table 1). The peptides with experimental *m*/*z* values of 1723.7 and 1886.5 Da were predicted to be segments of histatin-1, which are considered to be one major factor of the protective proteinaceous structure on the tooth surface (enamel pellicle), exhibiting antibacterial and antifungal activity [18, 34].

**Table 1:** Peptide sequences identified by LTQ-Orbitrap-MS among those differentially expressed in MALDI-TOF MS

Experimental *m*/*z* value	Peptide (SwissProt Code)	Peptide sequence	Theoretical *m*/*z* value
1370.4	Unknown peptide		
1723.7	sp|P15515| HUMAN histatin-1	REFPFYGDYGSNYL	1726.763
1851.4	sp|P02144| myoglobin	GHHEAEIKPLAQSHATK	1852.954
1886.5	sp|P15515| HUMAN histatin-1	REFPFYGDYGSNYLY	1889.826
2331.0	sp|P12273| prolactin-inducible protein	AAVIPIKNNRFYTIEILKVE	2330.336
2995.7	Unknown peptide		
3310.0	Unknown peptide		

Values reported here are the mono-charged ions of the peptides of interest for the study

*m*/*z* mass-to-charge ratio

### Western blotting results

**Fig. 4:**
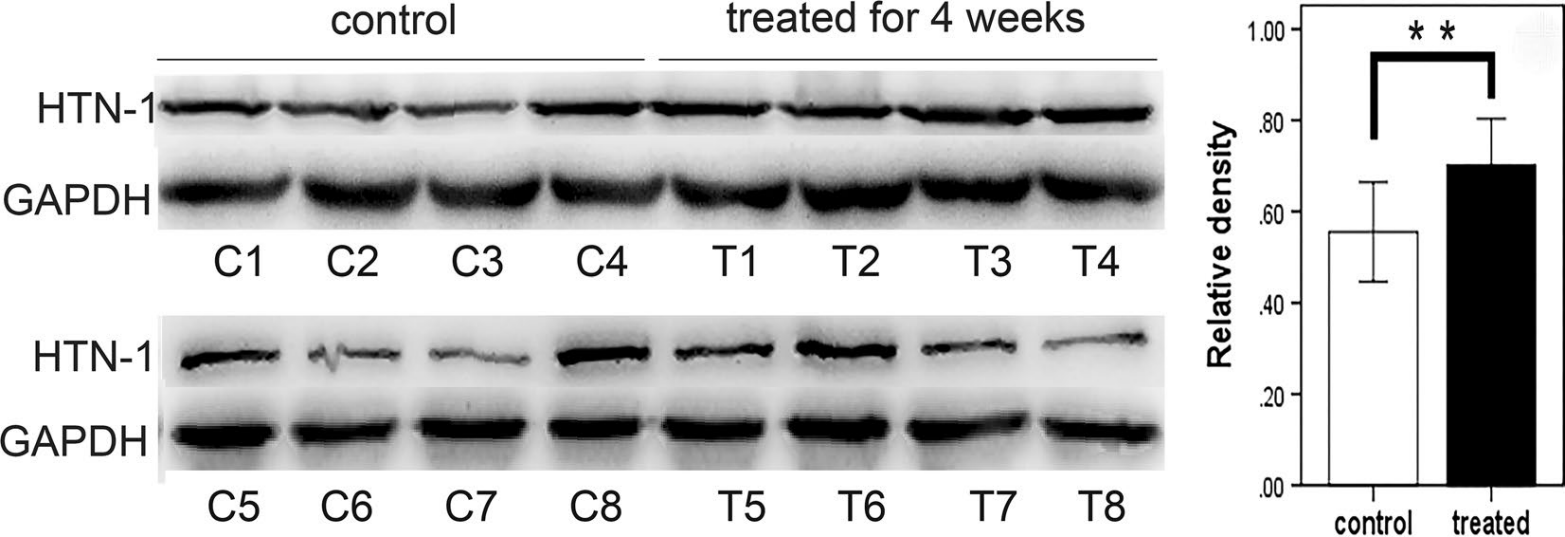
Salivary histatin-1 levels increased in children with s-ECC treated for 4 weeks. These levels were compared with before treatment. **a** Western blotting reveals the presence of histatin-1 in each salivary sample. **b** The relative density of the histatin-1 bands, revealing that levels of salivary histatin-1 are higher in children with s-ECC after treated for 4 weeks compared with before treatment  (**P < 0.01)

Sixteen stimulated whole saliva samples were collected from eight children with s-ECC (randomly chosen from the whole sample) before and after 4 weeks of treatment. The density of histatin-1 was divided by that of GAPDH for each sample to normalize the level of histatin-1. The levels of salivary histatin-1 were higher in children with s-ECC after treatment for 4 weeks compared with before treatment (*P* < 0.01) (Fig. 4).

## Discussion

Paediatric dental caries is a major public health problem, becoming the most common chronic infectious disease in childhood of great concern to parents and dentists [4, 6]. Saliva and its components play important roles in protecting oral structures. Although infection of *Streptococcus mutans*, inappropriate feeding and diet, and poor oral hygiene care are all causal factors, but endogenous factors, such as characteristics of saliva, may determine why some children develop caries while others do not [15, 35, 36].

In this study, we used proteomic technique to detect and discriminate peptide profiles in stimulated whole saliva from children aged 3–5 years with s-ECC compared with after 1 and 4 weeks of treatment. Conventional salivary proteomics studies mainly used 2-DE and SDS-PAGE, in which proteins are separated in terms of their molecular weight, followed by MS for identification. However, these strategies have several significant disadvantages, including complex procedures, the large sample size required, and that the informative low molecular weight peptides are likely outside their detection range [37].

Currently, the contribution of the different sources to whole saliva, and the evaluation of individual variability and physiological modifications have been investigated by top–down proteomic approaches, disclosing the faceted and complex profile of the human salivary proteome [38]. In a study, the whole saliva of 88 children aged between 0 and 48 months were analysed by a top–down platform based on RP-HPLC–ESI–MS, the result showed how the salivary proteome of human children evolves during infant development and concentrations of some salivary proteins (acidic proline-rich proteins, histatin-1, histatin-3, statherin, P-B peptide, and salivary cystatins) slowly increased with age, with different trends [39]. Top-down proteomic approaches were also used to detect biomarkers in some systemic diseases, such as schizophrenic and bipolar disorder [40], lung cancer [41], and chronic graft-versus-host disease [42].

In our study, we are using MB-based MALDI-TOF MS, which is an approach that overcomes some limitations of conventional salivary proteomics. MBs are micro-particles available with a range of different surface chemistries providing a choice of functionalities [43]. They are used for sample preparation (e.g., saliva, serum, plasma, urine, and cerebrospinal fluid) to enrich target low molecular weight and low-concentration peptides by their specific affinities [31]. In comparison with other time-consuming techniques in proteomics, the MB-based MALDI-TOF MS technique is rapid, high-throughput, and precise, with adequate resolution and sensitivity for the investigation of complex salivary samples [32]. It has also been used to detect and identify specific candidate biomarkers, not only in oral pathologies, such as periodontitis [44] and oral cancer [45], but also in systemic diseases, such as Sjögren’s syndrome [46], rheumatoid arthritis [47], and systemic lupus erythematosus [30].

In our results, the intensities of seven peaks (Figs. 2, 3a) differed significantly between the pre- and post-treatment s-ECC groups, indicating that this method can be used to analyse the peptide profiles in children with dental caries. We also found that several peptides were associated with having experienced caries, and might influence the onset and development of early childhood caries. We verified the diagnostic model established using four peptides (experimental *m*/*z* values: 1723.7, 1851.4, 2331.0, and 2995.7 Da) in other six cases and the results showed sensitivity and specificity of this model were both 83.3 %, indicating these salivary peptides should be potential candidate biomarkers of s-ECC. In the previous study by Si et al. [33], the findings revealed different composition of significantly expressed peptides between children with and without dental caries under a cross-sectional study design. The samples were from children in a different kindergarten, and dental caries in that study were not treated before analysis. Our present study was a longitudinal study with caries treatment, which was an entirely different design and could better obtain the potential biomarkers of caries treatment and experience. However, further studies are still needed if we would like to draw a full picture of diagnosis of dental caries, especially to explore the difference between untreated non-caries, untreated caries, well-treated caries and secondary caries.

Some previous studies have identified salivary peptides, including histatins, defensins, and proline-rich proteins (PRPs), may contribute to paediatric caries [18, 48, 49]. In this study, two peptides (experimental *m*/*z* values: 1723.7 and 1886.5 Da) were predicted to be segments of histatin-1 (Table 1), and their levels of expression were lower in the pre-treatment than post-treatment s-ECC group. However, the difference between their sequences were only one amino acid (tyrosine), indicating these two peptides might be homogenous. But on the other hand, this will provide much stronger evidence for histatin-1 as a candidate biomarker for dental caries.

Histatins are salivary proteins that have a high affinity for hydroxyapatite and contribute to the acquired enamel pellicle [50]. When histatins are adsorbed onto the enamel surface, they provide protection against acid aggression [51]. In this study, we collected 16 stimulated whole saliva samples from 8 children with s-ECC before and after 4 weeks of treatment (equal amounts of the total protein were used for each sample) who were randomly chosen from the whole sample, to perform western blotting assay in order to learn the difference of protein expression of histatin-1 between different time points before and after treatment of dental caries. Western blotting was performed in parallel with constant conditions, and we found that the levels of salivary histatin-1 were higher in children with s-ECC after treatment for 4 weeks compared with before treatment (*P* < 0.01), which was consistent with the tendency of expression of the two peptides with experimental *m*/*z* values of 1723.7 and 1886.5 during different period of treatment of dental caries in MALDI-TOF MS. In a study performed in 2005, high-performance liquid chromatography-mass spectrometry (HPLC–MS) was used to investigate the correlation between salivary peptides and dental caries in 20 samples, who were divided by dmfs index into two groups [18]. The results indicated strong correlations between high levels of phosphopeptides (PRP 1-3, histatin-1, and statherin) and the absence of dental caries. Results of the present study were consistent with it despite the different method applied. These above suggested that histatin-1 played a significant role in maintaining tooth integrity and protection against cariogenic bacteria, and might be associated with having experienced caries.

In addition, we found prolactin inducible protein (PIP) and myoglobin might also be candidate biomarkers of dental caries. PIP is a 17 kDa single polypeptide chain, known by various names due to its versatile nature and function in human reproductive and immune systems, which expressed in lacrimal, salivary, and sweat glands [52]. It is considered as a prognostic biomarker because of its over-expression in metastatic breast and prostate cancer [53, 54]. Moreover, its aspartyl-proteinase nature suggests its role in tumour progression [55, 56]. In saliva, PIP is a lipopolysaccharide-binding (LPS-binding) protein capable of binding to many bacteria living in oral cavity, including *A. actinomycetemcomitans*, *P. gingivalis* [57], *Gemella haemolysans*, *Gemella morbillorium*, *Streptococcus acidominimus*, *Streptococcus oralis*, *Streptococcus salivarius* and *Streptococcus parasanguinis* [58]. PIP may play an important role in the blockage of bacterial adhesion, contributing to pathogenesis and host immunity. We could only find very few related studies on the relationship between PIP and dental caries in recent years. In one study in 2014, magnetic beads and LTQ-Orbitrap hybrid Fourier transform mass spectrometry were used to identify the *Streptococcus mutans* lipoteichoic acid (Sm.LTA) binding proteins in the saliva of caries-free and caries human subjects. However, the results indicated that the expression of prolactin-inducible protein had no significant difference in both groups [59]. Meanwhile, myoglobin, one protein that was known to bind oxygen in myocardium and skeletal muscles, was not reported relevant to saliva and dental caries in previous studies. Further studies will be needed to explore the probable function of PIP and myoglobin in dental caries.

Although proteome profiling using MB-based MALDI-TOF MS together with LTQ-Orbitrap-MS is effective for the identification of saliva-specific peptides, some limitations are still remaining. First, identifying all the peaks was challenging as some peptides in the saliva may be secreted by epithelial cells of salivary glands and oral microorganisms, or may be produced by proteolysis or inflammatory factors associated with the body’s response to infection [60, 61], which might become confounding factors when searching for candidate diagnostic biomarkers of dental caries. Second, among the seven differentially expressed peptides, only four of them were identified successfully, while the other three had no matching results from LTQ-Orbitrap-MS. We speculate these peptides might have varying stability when using different MS methods, and hopefully these peptides can be identified if we could introduce other precise methods of peptidomics with less impact on peptide stability in future studies. Third, the bio-informative identification of these identified peptides were performed by searching human peptide and protein databases, indicating the authentic origination of these peptides may not fully match the identified sources present in the databases. There should also be some possibility that these peptides might be secreted by some microorganisms or other sources in saliva. This issue will not be fully resolved until the database is improved to become much more complete in the future, and we may have a look back on these peptides periodically as the database is ever-growing all the time. Fourth, as for histatin-1, due to the limitations of current methodology and dynamic transformation of histatin-1 in a variety of physiological, biochemical and metabolic processes, we were not able to detect the entire sequence of histatin-1 by mass spectrometry, though we had confirmed levels of salivary histatin-1 are higher in children with s-ECC after treatment for 4 weeks compared with before treatment by western blotting. In the future, with the development of research technologies and improvement of peptidomic methodology, we are looking forward to performing more in-depth studies on the intrinsic mechanisms between histatin-1 and the occurrence and development of dental caries. Last but not the least, the number of samples analysed was not quite enough, which may limit the validity of the results. Further studies in larger patient cohorts with blinded samples are required to confirm the usefulness of these diagnosis-oriented peptides, together with the function and structures of these novel candidate biomarkers. This will facilitate saliva proteomic profiling of s-ECC at the molecular level and yield a better understanding of s-ECC and its prevention.

## Conclusions

In summary, these results indicated that proteomics approaches can help discovery of new candidate biomarkers and identification of s-ECC-related proteins. The combination of 4 peptides here (experimental *m*/*z* values: 1723.7, 1851.4, 2331.0, and 2995.7 Da) may be a diagnostic indicator of the status of s-ECC. Besides, 2 differentially expressed peptides (experimental *m*/*z* values: 1723.7 and 1886.5), which might be probably homogenous, were predicted to originate from histatin-1, and histatin-1 was also identified as one candidate biomarker at protein level. In the future, caries risk of children is hopeful to be assessed by testing the specific peptides in saliva, which can be useful for drawing up individualised preventive strategies.

## Additional file

10.1186/s12967-016-0996-4 Detailed information of the participants and differentially expressed peptide peaks before and after caries treatment.

## Abbreviations

ANOVA: analysis of variance
BCA: bicinchoninic acid
CHCA: alpha-cyano-4-hydroxycinnamic acid
GAPDH: glyceraldehyde-3-phosphate dehydrogenase
HPLC-MS: high-performance liquid chromatography-mass spectrometry
IgG-HRP: immuno-globin G-horseradish peroxidase
KNN: K nearest-neighbour
LPS: lipopolysaccharide
LTQ-Orbitrap-MS: linear ion trap-orbitrap-mass spectrometry
MALDI-TOF MS: matrix-assisted laser desorption/ionization time-of-flight mass spectrometry
MB: magnetic bead
Nano-LC/ESI–MS/MS: nano-liquid chromatography-electrospray ionization-tandem mass spectrometry
PIP: prolactin inducible protein
PRP: proline-rich protein
SDS-PAGE: sodium dodecyl sulfate polyacrylamide gel electrophoresis
s-ECC: severe early childhood caries
PVDF: polyvinylidene fluoride
Sm.LTA: *Streptococcus mutans* lipoteichoic acid
TBS: tris-buffered saline
TFA: α-cyano-4-hydroxycinnamic acid
WCX MB: weak cation exchange magnetic bead
