# Supplementary material for: Salivary peptidome profiling for diagnosis of severe early childhood caries
Source: J Transl Med. 2016 Aug 15;14:240. doi: 10.1186/s12967-016-0996-4 (PMC4986381; doi:10.1186/s12967-016-0996-4)
Supplement: Supplementary file 1 — 10.1186/s12967-016-0996-4 Detailed information of the participants and differentially expressed peptide peaks before and after caries treatment. [file 12967_2016_996_MOESM1_ESM.docx]

**Salivary peptidome profiling for diagnosis of severe early childhood caries**

***Additional File 1.*** *Detailed information of the participants and differentially expressed peptide peaks before and after caries treatment*

Xiangyu Sun ^1,#^, Xin Huang ^1,#^, Xu Tan ^1,2,#^, Yan Si ^1^, Xiaozhe Wang ^1^, Feng Chen ^3,**^, Shuguo Zheng ^1,*^

1 Department of Preventive Dentistry, Peking University School and Hospital of Stomatology, National Engineering Laboratory for Digital and Material Technology of Stomatology, Beijing Key Laboratory of Digital Stomatology, Beijing 100081, PR China.

2 Stomatological Hospital of Guizhou Medical University, Guiyang 550004, PR China

3 Central Laboratory, Peking University School and Hospital of Stomatology, National Engineering Laboratory for Digital and Material Technology of Stomatology, Beijing Key Laboratory of Digital Stomatology, Beijing 100081, PR China.

^#^ Xiangyu Sun, Xin Huang and Xu Tan contribute equally to this article.

* Corresponding author.

Address: Department of Preventive Dentistry, Peking University School and Hospital of Stomatology, National Engineering Laboratory for Digital and Material Technology of Stomatology, Beijing Key Laboratory of Digital Stomatology, 22 Zhongguancun Avenue South, Haidian District, Beijing 100081, PR China.

Email: [zhengsg86@gmail.com](mailto:zhengsg86@gmail.com); Tel: +86 10 82195510; Fax: +86 10 62173404.

** Co-corresponding author.

Address: Central Laboratory, Peking University School and Hospital of Stomatology, National Engineering Laboratory for Digital and Material Technology of Stomatology, Beijing Key Laboratory of Digital Stomatology, 22 Zhongguancun Avenue South, Haidian District, Beijing 100081, PR China.

Email: [moleculecf@gmail.com](mailto:moleculecf@gmail.com)

**Additional Table 1. Information of the children recruited in the study**

| Stage | Sample size | Age | | Sex proportion  (Male : Female) |
| --- | --- | --- | --- | --- |
|  |  | Mean | SD |  |
| Modelling stage | 10 | 4.7 | 0.5 | 7:3 |
| Validation stage | 6 | 4.8 | 0.6 | 4:2 |

**Additional Table 2. Comparison of the 81 peptide peaks detected simultaneously in all the three time points**

| *m/z* | *PTTA(f)* | *P-KWTest* | *PAD_1* | *PAD_2* | *PAD_3* |
| --- | --- | --- | --- | --- | --- |
| **1370.4** | <1.00*10^-6^ | **2.35*10^-4^** | 0.231 | 0.001 | 0.052 |
| **1723.7** | **<1.00*10^-6^** | <1.00*10^-6^ | 0.001 | 0.008 | 0.001 |
| **1851.4** | 4.81*10^-5^ | **4.41*10^-5^** | 0.246 | 0.041 | 0.007 |
| **1886.5** | 1.01*10^-4^ | **6.72*10^-4^** | 0.023 | 0.109 | 0.050 |
| **2331.0** | **2.75*10^-6^** | 2.12*10^-4^ | 0.001 | 0.005 | 0.003 |
| **2995.7** | **<1.00*10^-6^** | <1.00*10^-6^ | 0.007 | 0.001 | 0.002 |
| **3310.0** | <1.00*10^-6^ | **2.72*10^-5^** | 0.387 | 0.004 | 0.106 |

| *m/z* | *PTTA(f)* | *P-KWTest* | *PAD_1* | *PAD_2* | *PAD_3* |
| --- | --- | --- | --- | --- | --- |
| 1027.4 | 0.025 | 0.015 | 0.256 | 0.001 | 0.013 |
| 1048.8 | 0.017 | 0.018 | 0.500 | 0.001 | 0.295 |
| 1058.4 | 0.460 | 0.831 | 0.383 | 0.140 | 0.003 |
| 1074.2 | 0.195 | 0.302 | 0.361 | 0.105 | 0.020 |
| 1090.0 | 0.434 | 0.580 | 0.014 | 0.063 | 0.004 |
| 1112.1 | 0.096 | 0.141 | 0.057 | 0.001 | 0.008 |
| 1184.9 | 0.071 | 0.138 | 0.067 | 0.021 | 0.146 |
| 1211.1 | 0.145 | 0.195 | 0.496 | 0.045 | 0.038 |
| 1227.3 | 0.062 | 0.019 | 0.001 | 0.004 | 0.001 |
| 1246.2 | 0.274 | 0.695 | 0.121 | 0.021 | 0.368 |
| 1268.5 | 0.093 | 0.182 | 0.241 | 0.048 | 0.002 |
| 1284.3 | 0.214 | 0.247 | 0.074 | 0.094 | 0.350 |
| 1300.1 | 0.066 | 0.166 | 0.306 | 0.073 | 0.500 |
| 1322.1 | 0.409 | 0.624 | 0.067 | 0.034 | 0.002 |
| 1348.9 | 0.531 | 0.538 | 0.037 | 0.307 | 0.112 |
| 1382.1 | 0.136 | 0.001 | 0.001 | 0.066 | 0.050 |
| 1390.4 | 0.853 | 0.772 | 0.001 | 0.038 | 0.622 |
| 1400.1 | 0.553 | 0.725 | 0.001 | 0.003 | 0.008 |
| 1425.4 | 0.003 | 0.341 | 0.001 | 0.001 | 0.412 |
| 1472.5 | 0.197 | 0.076 | 0.275 | 0.001 | 0.379 |
| 1494.3 | 0.004 | 0.035 | 0.139 | 0.087 | 0.104 |
| 1510.1 | 0.789 | 0.872 | 0.046 | 0.073 | 0.341 |
| 1523.7 | 0.090 | 0.113 | 0.023 | 0.001 | 0.001 |
| 1552.9 | 0.029 | 0.003 | 0.001 | 0.005 | 0.007 |
| 1687.8 | 0.154 | 0.026 | 0.279 | 0.001 | 0.251 |
| 1708.2 | 0.014 | 0.001 | 0.001 | 0.002 | 0.003 |
| 1747.9 | 0.059 | 0.037 | 0.024 | 0.001 | 0.045 |
| 1767.1 | 0.042 | 0.006 | 0.003 | 0.007 | 0.179 |
| 1775.4 | 0.757 | 0.787 | 0.001 | 0.003 | 0.216 |
| 1822.4 | 0.194 | 0.005 | 0.001 | 0.049 | 0.049 |
| 1832.5 | 0.370 | 0.888 | 0.004 | 0.010 | 0.249 |
| 1894.3 | 0.499 | 0.879 | 0.093 | 0.530 | 0.005 |
| 1903.1 | 0.030 | 0.041 | 0.091 | 0.001 | 0.210 |
| 1915.0 | 0.517 | 0.991 | 0.001 | 0.001 | 0.050 |
| 1931.9 | 0.379 | 0.291 | 0.505 | 0.123 | 0.035 |
| 1942.1 | 0.319 | 0.554 | 0.183 | 0.001 | 0.009 |
| 1952.6 | 0.259 | 0.032 | 0.001 | 0.001 | 0.003 |
| 2001.6 | 0.188 | 0.274 | 0.001 | 0.056 | 0.100 |
| 2028.8 | 0.009 | 0.050 | 0.001 | 0.202 | 0.307 |
| 2080.3 | 0.894 | 0.191 | 0.005 | 0.001 | 0.003 |
| 2106.5 | 0.955 | 0.345 | 0.002 | 0.294 | 0.078 |
| 2115.2 | 0.669 | 0.792 | 0.027 | 0.151 | 0.039 |
| 2184.2 | 0.706 | 0.413 | 0.001 | 0.062 | 0.001 |
| 2197.5 | 0.372 | 0.332 | 0.001 | 0.001 | 0.001 |
| 2216.8 | 0.682 | 0.606 | 0.537 | 0.387 | 0.245 |
| 2231.7 | 0.322 | 0.473 | 0.001 | 0.036 | 0.062 |
| 2243.9 | 0.365 | 0.365 | 0.064 | 0.021 | 0.088 |
| 2363.6 | 0.994 | 0.443 | 0.011 | 0.001 | 0.009 |
| 2401.8 | 0.024 | 0.083 | 0.001 | 0.017 | 0.443 |
| 2447.5 | 0.141 | 0.131 | 0.058 | 0.043 | 0.005 |
| 2491.2 | 0.472 | 0.302 | 0.006 | 0.056 | 0.002 |
| 2527.3 | 0.734 | 0.489 | 0.003 | 0.002 | 0.369 |
| 2573.6 | 0.202 | 0.584 | 0.001 | 0.001 | 0.008 |
| 2603.8 | 0.763 | 0.580 | 0.500 | 0.111 | 0.033 |
| 2952.8 | 0.933 | 0.801 | 0.001 | 0.035 | 0.621 |
| 3011.5 | 0.113 | 0.007 | 0.001 | 0.211 | 0.048 |
| 3019.2 | 0.128 | 0.093 | 0.001 | 0.142 | 0.006 |
| 3121.9 | 0.895 | 0.678 | 0.102 | 0.010 | 0.301 |
| 3142.9 | 0.743 | 0.497 | 0.279 | 0.029 | 0.047 |
| 3162.8 | 0.102 | 0.159 | 0.001 | 0.016 | 0.004 |
| 3367.5 | 0.623 | 0.055 | 0.001 | 0.001 | 0.005 |
| 3402.3 | 0.081 | 0.128 | 0.028 | 0.033 | 0.042 |
| 3485.3 | 0.168 | 0.038 | 0.002 | 0.001 | 0.004 |
| 3502.7 | 0.545 | 0.744 | 0.105 | 0.032 | 0.373 |
| 3529.3 | 0.473 | 0.642 | 0.001 | 0.106 | 0.752 |
| 3781.9 | 0.008 | 0.012 | 0.114 | 0.001 | 0.280 |
| 3950.3 | 0.210 | 0.119 | 0.251 | 0.056 | 0.003 |
| 4165.2 | 0.003 | 0.005 | 0.073 | 0.001 | 0.206 |
| 4174.4 | 0.003 | 0.011 | 0.058 | 0.001 | 0.236 |
| 4489.9 | 0.615 | 0.624 | 0.001 | 0.001 | 0.010 |
| 4520.9 | 0.248 | 0.302 | 0.021 | 0.001 | 0.108 |
| 4675.9 | 0.011 | 0.003 | 0.001 | 0.004 | 0.005 |
| 4785.8 | 0.059 | 0.040 | 0.001 | 0.001 | 0.002 |
| 4813.7 | 0.319 | 0.458 | 0.001 | 0.001 | 0.001 |

*P*<0.01 was considered as threshold of statistical significance.

*PTTA(f)*, *P* value of ANOVA. *P-KWTest*, *P* value of Kruskal-Wallis test.

Which *P* value was used for the peptide depended on the results of normality tests:

*PAD_1*, normality test of s-ECC untreated.

*PAD_2*, normality test of s-ECC treated for 1 week.

*PAD_3*, normality test of s-ECC treated for 4 weeks.

**Additional Table 3. Significance, abundance values and tendency of the seven peptides differentially expressed among the three time points**

| *m/z* | *P*-value | s-ECC  untreated | s-ECC treated  for 1 week | s-ECC treated  for 4 weeks | Tendency |
| --- | --- | --- | --- | --- | --- |
| 1370.4 | 2.35*10^-4^ | 305.1 | 258.4 | 164 | **↓** |
| 1723.7 | <1.00*10^-6^ | 206 | 804.3 | 1134.5 | ↑ |
| 1851.4 | 4.41*10^-5^ | 308 | 400.6 | 505.2 | ↑ |
| 1886.5 | 6.72*10^-4^ | 119.7 | 219.3 | 334.7 | ↑ |
| 2331.0 | 2.75*10^-6^ | 308.3 | 407.7 | 545.3 | ↑ |
| 2995.7 | <1.00*10^-6^ | 80.8 | 320.6 | 409.3 | ↑ |
| 3310.0 | 2.72*10^-5^ | 336.2 | 225.6 | 95 | **↓** |
